# Spontaneous Heterotopic Pregnancy: Dual Case Report and Review of Literature

**DOI:** 10.1155/2016/2145937

**Published:** 2016-06-19

**Authors:** Annika Chadee, Shadi Rezai, Catherine Kirby, Ekaterina Chadwick, Sri Gottimukkala, Abraham Hamaoui, Vasiliy Stankovich, Theodore Hale, Hamid Gilak, Mohammad Momtaz, Harvey Sasken, Cassandra E. Henderson

**Affiliations:** ^1^Department of Obstetrics and Gynecology, Lincoln Medical and Mental Health Center, Bronx, NY 10451, USA; ^2^West Virginia School of Osteopathic Medicine (WVSOM), Lewisburg, WV 24901, USA; ^3^Department of Pathology, Lincoln Medical and Mental Health Center, Bronx, NY 10451, USA

## Abstract

*Introduction.* Heterotopic pregnancy is a rare complication usually seen in populations at risk for ectopic pregnancy or those undergoing fertility treatments. It is a potentially dangerous condition occurring in only 1 in 30,000 spontaneous pregnancies. With the advent of Assisted Reproduction Techniques (ART) and ovulation induction, the overall incidence of heterotopic pregnancy has risen to approximately 1 in 3,900 pregnancies. Other risk factors include a history of pelvic inflammatory disease (PID), tubal damage, pelvic surgery, uterine Mullerian abnormalities, and prior tubal surgery. Heterotopic pregnancy is a potentially fatal condition, rarely occurring in natural conception cycles. Most commonly, heterotopic pregnancy is diagnosed at the time of rupture when surgical management is required.* Case.* This paper represents two cases of heterotopic pregnancies as well as a literature review.* Conclusion.* Heterotopic pregnancy should be suspected in patients with an adnexal mass, even in the absence of risk factors. Clinicians must be alert to the fact that confirming an intrauterine pregnancy clinically or by ultrasound does not exclude the coexistence of an ectopic pregnancy. A high index of suspicion in women is needed for early and timely diagnosis, and management with laparotomy or laparoscopy can result in a favorable and successful obstetrical outcome.

## 1. Introduction

Heterotopic pregnancy is the simultaneous coexistence of an intrauterine and an extrauterine gestation [1–6]. It is a rare and potentially dangerous condition occurring in only 1 in 30,000 spontaneous pregnancies [7–12]. With the advent of Assisted Reproduction Techniques (ART) [12–14] and ovulation induction, the overall incidence of heterotopic pregnancy has risen to approximately 1 in 3,900 pregnancies [15]. However, obstetricians and emergency medicine physicians are unlikely to consider this diagnosis as a part of the differential in cases presenting with abdominal pain and vaginal bleeding.

Transvaginal ultrasound is the key to diagnosing heterotopic pregnancy [8, 16]. However, it continues to have a low sensitivity because the diagnosis is often missed or overlooked [17, 18]. Therefore the diagnosis is often delayed leading to serious consequences.

Surgical intervention plays a key role in the management of heterotopic pregnancy [19]. The goal is to remove the ectopic pregnancy without jeopardizing the intrauterine pregnancy [20]. Laparoscopic salpingectomy is the standard surgical approach of heterotopic pregnancy. Other management options mentioned in the literature include local injection of potassium chloride, hyperosmolar glucose, or methotrexate into the sac under ultrasound guidance followed by aspiration of the ectopic pregnancy [21]. This paper represents two cases of heterotopic pregnancies as well as review of literature.

## 2. Presentation of the Case Number 1

Patient is a 34-year-old Hispanic female, G3P1011 at 9 weeks and four days by last menstrual period, consistent with a 9 weeks and three days sonogram, presented to the emergency room complaining of vaginal spotting, lower abdominal pain, and nausea. She denied any leakage of fluid, urinary symptoms, fever, chills, dizziness, palpations, or any other symptoms.

The patient had a history of a prior hospitalization for pelvic inflammatory disease (PID) with a retained intrauterine device (IUD) about seven years priorly. Her IUD was removed in the operating room. Her cultures at that time grew* Actinomyces*,* Streptococcus viridans*, and coagulase negative* Staphylococcus* and she was treated with Doxycycline and Ceftriaxone. Patient did not have any other past medical, surgical, or family history.

Upon presentation to emergency department, the patient was noted to be pale and her abdomen was mildly distended and was tender more on the right side than left. On vaginal exam, there was no active bleeding or leakage of fluid. The cervix was found to be closed, long, and posterior, with cervical motion tenderness and bilateral adnexal tenderness. No adnexal masses were appreciated. There were no other pertinent significant physical findings.

The patient's blood type was A Rh positive, with Beta-HCG value of 146,864 mIU/mL, which correlates with a gestation of six to eight weeks. The progesterone value was 28.52 ng/mL, appropriate for an early first trimester pregnancy. The patient's initial hemoglobin was 10.3 g/dL, hematocrit of 30.6%, MCV of 89.1 fL, and RDW of 14.1%. Follow-up hemograms showed a continuous drop in the hemoglobin/hematocrit through 8.7/26.3 to 7.6/22.8.

**Figure 1:**
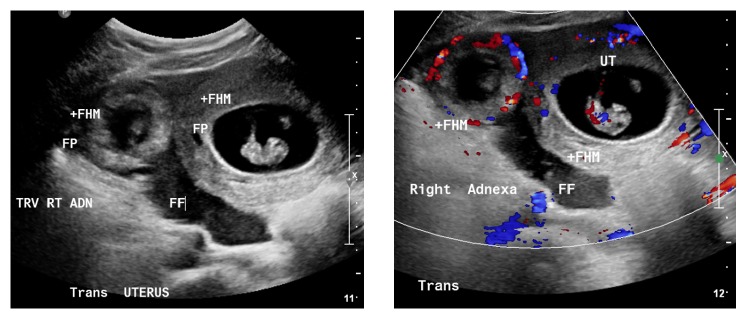
Patient number 1. Pelvic ultrasound showing the intrauterine and extrauterine/tubal ectopic pregnancies, both with fetal pole (FP), fetal heart rates (+FHM) present, and free fluid (FF) in the peritoneal cavity.

Pelvic ultrasound (Figure 1) revealed a single live intrauterine pregnancy of 8 weeks and 4 days with a fetal heart rate of 188 beats per minute (BPM) and an ectopic pregnancy was seen in the right adnexa of 8 weeks and 0 days with a fetal heart rate of 183 bpm. A large amount of free fluid was present, consistent with the acute blood loss noted in the dropping hemoglobin/hematocrit.

**Figure 2:**
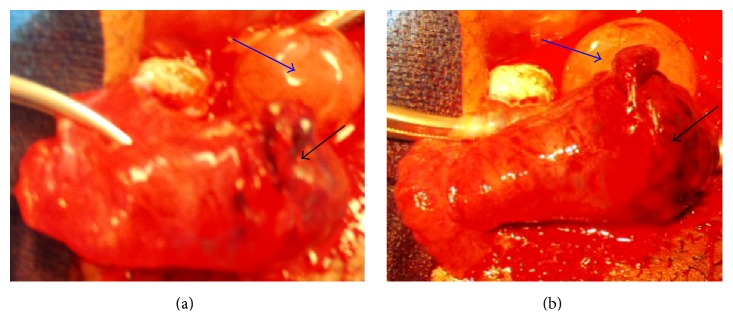
Patient number 1. Operative finding showing a ruptured right ectopic pregnancy (black arrow) with an ovarian corpus luteal cyst (blue arrow).

An exploratory laparotomy was performed revealing an approximately 5 cm × 3 cm ruptured ectopic pregnancy at the ampulla of the right fallopian tube, as well as an intact ovarian corpus luteal cyst (Figure 2). A right salpingectomy and evacuation of 300 cc of hemoperitoneum was performed. Histopathology confirmed the diagnosis of an ectopic pregnancy. The fetal heart rate of the intrauterine pregnancy was present before and after surgery. The hospital course was uneventful and the patient struggled with the decision whether to keep the intrauterine pregnancy or not. Despite the fact that the intrauterine pregnancy was progressing, the patient was unconvinced and opted for termination of the intrauterine pregnancy via dilation and curettage on postoperative day fourteen.

## 3. Presentation of the Case Number 2

Patient is 32 years old, G1P0 at 7 6/7 weeks by last menstrual period of 1/22/14, who initially presented in the ED on 3/16/14 with complaining of vaginal bleeding, and she was diagnosed with complete abortion. On clinic follow-up visit on 3/18/14, Beta-HCG level was 22995 mIU/mL increasing from 20571 mIU/mL on 3/16/14. Patient denied any complaints including any vaginal bleeding or abdominal pain on follow-up visit, and she was hemodynamically stable.

**Figure 3:**
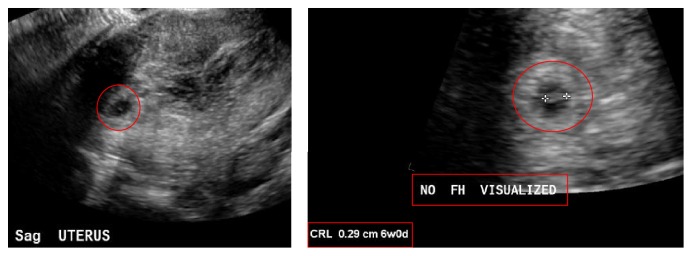
Patient number 2. Obstetric ultrasound on 3/2/2014: intrauterine gestational sac seen. There is adjacent heterogeneity suggesting subchorionic hemorrhage. No fetal cardiac activity detected. Fetal pole measures approximately 2.9 mm.

An obstetric ultrasound on 3/2/14 (Figure 3) revealed intrauterine gestational sac and subchorionic hemorrhage, as well as a fetal pole measuring approximately 2.9 mm with no fetal cardiac activity at that time (Figure 3).

Repeat ultrasound on 3/16/14 showed the previously described intrauterine gestation with fetal pole is no longer definitely seen. A collapsed sac however may be seen at the lower uterine segment.

Third ultrasound on 3/18/14 showed an echotexture uterus measuring 12 × 4.6 × 6.5 cm. The endometrium remained markedly thickened and heterogeneous containing multiple cystic components. It measured up to 3.5 cm in thickness. There was increased vascularity towards the fundus; on the left, there was a hypoechoic structure measuring 2.5 × 2.5 × 2.3 cm with mild vascularity. This was not clearly delineated on prior imaging. There was no fluid in the cul-de-sac.

*Beta-HCG Trend (mIU/mL).* It was 2484 (2/26/14), 4211 (3/2/14), 20571 (3/16/14), 22995 (3/18/15), and 3921 (3/21/14).

**Figure 4:**
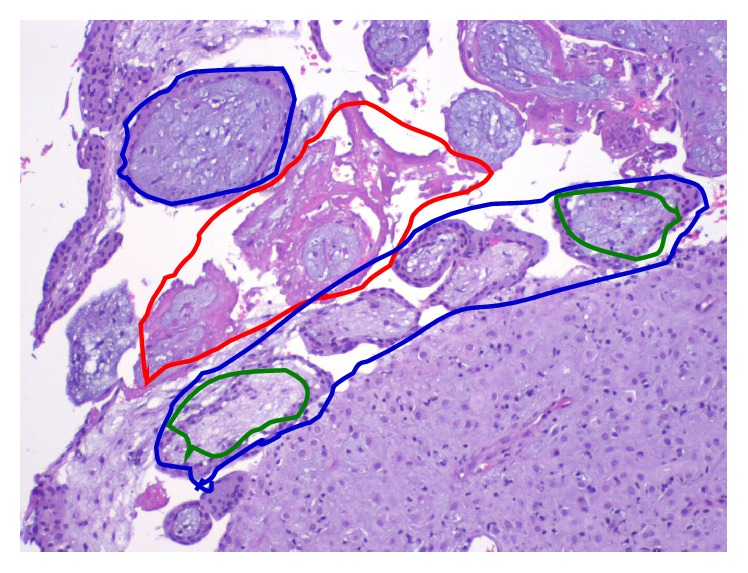
Pathology for patient 2. Specimen 1: endometrium and product of conception (POC) from D&E for VTOP. Green circles: decidua and chorionic villi with viable syncytiotrophoblast; blue circles (purple color): degenerating villi; red circles (pink color): fibrin or degenerated villi.

Patient underwent suction dilatation and curettage (D&C) for incomplete abortion. Moderate amount of product of conception (POC) was collected during D&C, which pathologic examination showed to be decidua and chronic villi (Figure 4).

Intraoperative bedside sonogram was done after D&C and revealed complete evacuation of products of conception (POC) from uterus with no free fluid; however, there was still a hypoechoic structure along the fundus of the uterus.

**Figure 5:**
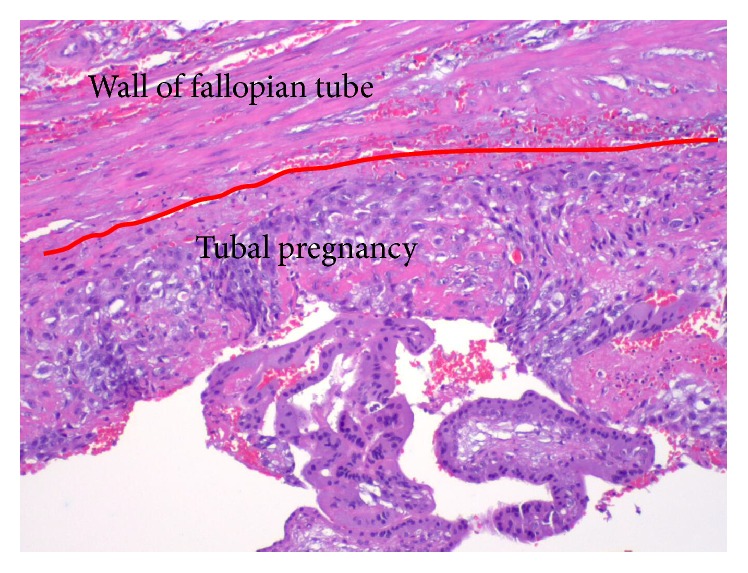
Pathology for patient 2. Specimen 2: left tubal ectopic pregnancy from laparoscopy. Tubal pregnancy: slide from fallopian tube, showing the wall of fallopian tube and a tubal pregnancy with decidua, chorionic villi, and syncytiotrophoblast inside the fallopian tube, confirming the ectopic pregnancy in the fallopian tube and therefore heterotopic pregnancy.

Laparoscopic excision of left corneal ectopic pregnancy with mini-laparotomy for specimen retrieval was done and an approximately 4 cm × 4 cm left corneal ectopic pregnancy was excised, which was also sent to pathology, and pathologic examination showed decidua and chronic villi (Figure 5) and therefore diagnosis of heterotopic pregnancy was confirmed. Patient had uncomplicated hospital stay and was discharged on postoperative day 3.

## 4. Discussions

When initially seen in the emergency room, the differentials included ectopic pregnancy, abortion, and ovarian torsion. However, the transvaginal ultrasound was instrumental in revealing the correct diagnosis. In this case, the patient had a history of having an IUD with pelvic inflammatory disease, which possibly increased her risk of having an ectopic pregnancy [15, 22]. When intrauterine pregnancy has already been established, the differential diagnosis of heterotopic pregnancy is even more commonly missed [23]. In this case the diagnosis of heterotopic pregnancy was accurately made with the use of transvaginal ultrasound, which allowed for timely diagnosis and management before grave consequences occurred. The ectopic pregnancy was ruptured and was diagnosed promptly by the presence of free fluid intraperitoneally in a background of a dropping hemoglobin level [24]. Intraoperatively, the ruptured right ectopic pregnancy was readily noted with the confirmation of a simultaneous intrauterine pregnancy.

Other case reports of heterotopic pregnancies have been reported.  Table 1 reviews the details of some of these cases.

## 5. Conclusion

Clinicians should always keep heterotopic pregnancy in the differential diagnosis in a reproductive patient with abdominal pain and signs or symptoms of ectopic pregnancy [25, 26]. They must be alert to the fact that confirming an intrauterine pregnancy clinically or by ultrasound does not exclude the coexistence of an ectopic pregnancy [27]. A high index of suspicion in women is needed for early and timely diagnosis, and management with laparotomy or laparoscopy can result in a favorable successful obstetrical outcome [28, 29]. Heterotopic pregnancy is possible with natural conception and the survival of the intrauterine fetus is feasible [9, 30, 31].

## Figures and Tables

**Table 1:** Summary of case reports.

Author	Patient	Presentation	Case details	Outcome
Fatema et al. [32]	38 years old G7P3A3	Abdominal pain and vomiting	Ruptured fallopian tube, initially misdiagnosed as appendicitis	Discharged but had a miscarriage 12 days later
S. K. Shetty and A. K. Shetty [24]	26 years old primigravida	Severe abdominal pain	Right sided ruptured fallopian tube	Started progesterone and carried intrauterine pregnancy to term
Simsek et al. [33]	37 years old	Abdominal pain	Ruptured fallopian tube with 2 intrauterine fetuses	Underwent laparotomy and carried intrauterine pregnancy to term
